# Fault Diagnosis and Handling of the Two-Dimensional Tracking Servo System for Space

**DOI:** 10.1155/2022/8174674

**Published:** 2022-06-22

**Authors:** Zelong Ma, Qinglei Zhao, Shuxin Wang

**Affiliations:** Changchun Institute of Optics, Fine Mechanics and Physics, Chinese Academy of Sciences, Changchun 130033, China

## Abstract

Aiming at the on-orbit safety problem of the two-dimensional tracking servo system for space, based on ant colony optimization (ACO) and expert systems, this paper proposes a fault diagnosis and handling strategy of the two-dimensional (2D) tracking servo system for space. Specifically, a fault library was established for the tracking servo system according to field observations. The ACO was called to optimize the classification of fault features. In addition, sensitivity and specificity were defined to evaluate the classification performance of the diagnosis rules found by the ant colony, aiming to suppress the redundant information in fault diagnosis, reduce the rows of the in-orbit codes, and improve the accuracy of in-orbit fault diagnosis. Meanwhile, the diagnosis information will be processed by the expert system, such that the servo system can work safely and stably in orbit, and less number of in-orbit computing resources were used. Simulation results show that the proposed strategy achieves highly precise and reliable diagnosis.

## 1. Introduction

The existing fault diagnosis techniques can be divided into three categories: those based on analytical models, those based on signal processing, and those based on knowledge. In recent years, artificial intelligence (AI)-based data mining techniques have been widely applied in the fault detection and diagnosis of complex systems, including neural networks, support vector machine (SVM), rough set, and intelligent optimization algorithms. These techniques are often combined to achieve better results [1–9].

Ant colony optimization (ACO) was proposed by Dorigo et al. [10, 11] by mimicking the foraging behavior of ants in nature, the group search for the shortest path to food. Parpinelli et al. introduced the ACO to medical diagnosis and proved that the algorithm can achieve higher classification accuracy with simpler rules than decision tree classifier Chance Node 2 (CN2).

Fuzzy reasoning, which imitates the way of human thinking in handling fuzzy information, is very suitable for processing nonlinear time-varying problems. The fuzziness of a system is proportional to the complexity of the system structure. Fuzzy reasoning can effectively deal with highly fuzzy systems.

With the boom of space tracking and control, the tracking servo system has been introduced to the field of aerospace, aiming to meet the growing requirement on the accuracy of in-orbit target tracking. Typically, the tracking servo system consists of a tacking turntable and an optical pointing system. This optical, mechanical, and electrical integrated control system can realize various functions, such as scanning, pointing, tracking, and measurement. It has been widely applied in earth observation, astronomical observation, space laser communication, target tracking, and deep space detection. However, these fault diagnosis and processing methods have not been applied to aerospace systems at present.

Although tracking servo system is widely used in ground and aerospace sectors, the two-dimensional (2D) tracking servo system for space has many unique features. The state of ground or aerospace servo control system is visible in real time. When it comes to the space, however, the ground control centers cannot receive satellite data in real time or in small intervals, owing to the narrow communication bandwidth of the star link and the limited communication time. Two problems would therefore arise:

1. If the tracking servo system fails, the failure cannot be solved timely through manual intervention, because it is impossible to download satellite data in real time.
2. Due to the long intervals between the data, the useful fault information is often not downloaded, making it difficult to correctly diagnose the failure. Any failure of the tracking servo system, as a movable part of the satellite, may threaten the operation of the entire satellite and even put the life of astronauts to jeopardy.

Therefore, the 2D tacking servo system for space needs an in-orbit autonomous strategy for fault diagnosis and handling. The strategy should be able to monitor, save, and download the real-time working state of the servo system. Once any failure occurs, the strategy must quickly pinpoint the fault source via fault diagnosis and take proper measures to restore the safety of the servo system.

Targeting a servo system for space, this paper firstly establishes a fault library and then calls ant colony optimization (ACO) to find high-precision fault diagnosis rules, which contain relatively few conditions. When the servo system fails, the current fault phenomenon is diagnosed to find the fault source according to the fault diagnosis rules. According to the information of the fault source, the fault was handled through fuzzy reasoning to ensure the working safety of the servo system.

## 2. Background

### 2.1. 2D Tracking Servo System for Space

**Figure 1:**
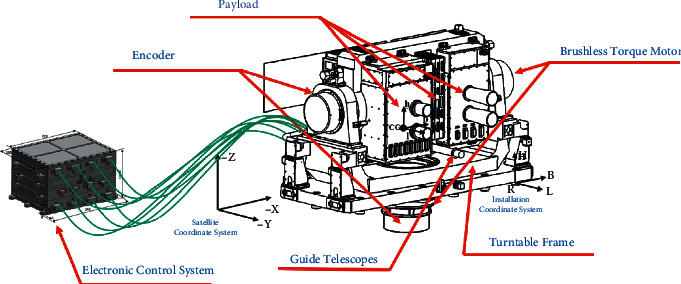
Sketch map of the 2D tracking servo system for space.

As shown in Figure 1, the 2D tracking servo system for space mainly consists of a turntable frame, guide telescopes, an encoder, a brushless torque motor, and an electronic control system. Among them, the guide telescopes, encoder, brushless torque motor, and effective payload are integrated onto the turntable frame. The electronic control system is connected to that frame via cables.

**Figure 2:**
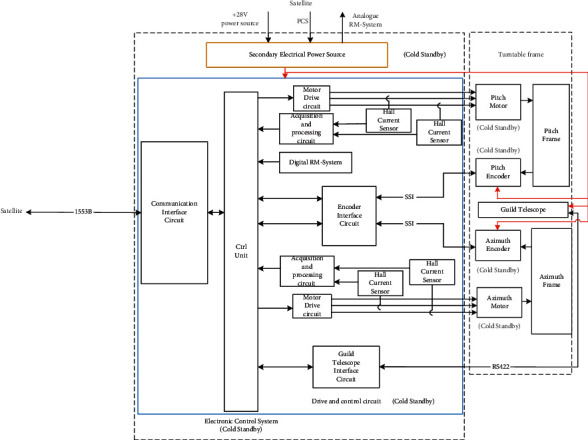
Sketch map of the communication of the 2D tracking servo system for space.

As shown in Figure 2, the electronic control system receives commands from the satellite platform and provides electricity and communication supports to the guide telescopes and encoder, both of which are position sensors. The encoder feeds back the positions of the two axes of the turntable to the electronic control system via serial communication, while the guide telescopes feed back the miss distance of the target. Through calculation, the electronic control system controls the brushless torque motor to drive the turntable. To improve reliability, cold standby is adopted for the electronic control system, encoder, and brushless torque motor.

In summary, the 2D tracking servo system for space is a highly compact and complex integrated system. The system failures are often homologous, involving many attributes. If a diagnosis program is prepared for every fault, the system will contain too many complex software codes. Thus, this paper proposes an intelligent classification algorithm based on the ACO and relies on the algorithm to find high-precision fault diagnosis rules, which contain relatively few conditions. Then, software codes were compiled following the found rules. When the system is in orbit, any fault detected would be handled immediately in orbit by the expert system, and then the fault information would be downloaded, waiting to be further processed on the ground.

### 2.2. Fault Library

The in-orbit working features of the 2D tracking servo system were defined as a vector of fault features *E*=(*e*_1_, *e*_2_, *e*_3_,…,*e*_11_), where *e*_1_–*e*_11_ refer to position state, velocity state, current state, torque state, motor temperature state, tracking precision state, communication state, analog/digital (AD) signal collection state, encoder state, guide telescope state, and single-event upset (SEU) states, respectively. The first ten states are fault phenomena that can be detected in real time in orbit. To prevent the SEU, all telemetered values were not encoded by single-byte encoding.  Table 1 lists the relationship between telemetered values and fault phenomena.

According to ground debugging and testing experience, this paper establishes a library for the correspondence between the fault phenomena and fault sources of the 2D tracking servo system for space, based on the telemetered values. However, the same fault source may correspond to multiple fault phenomena, and each fault may involve various features. Thus, it is no easy task to diagnose system failures. To solve the problem, this paper proposes an intelligent classification algorithm based on the ACO, and establishes high-precision diagnosis rules for fault source positioning, which contains relatively few conditions. These rules reduce the complexity of in-orbit software codes and enhance the autonomous fault diagnosis ability of the servo system. In addition, the expert system was employed to autonomously handle the diagnosed faults in orbit in real time, making the in-orbit operation of the servo system more secure and stable.

### 2.3. ACO-Based Design of Fault Diagnosis Plan

The ACO is a heuristic intelligent bionic optimization algorithm. The advantages of the algorithm include positive feedback and parallel distributed computing. In nature, the ant colony looks for food by the following principle: all ants leave from the nest and release pheromones along the way. The amount of pheromone on a path increases with the number of ants passing through that path. An ant prefers the path with a relatively high pheromone level. As a result, the pheromone level of high-quality paths will gradually rise, while that of poor-quality paths will gradually drop, due to pheromone volatilization. Eventually, the whole colony will find the best path to the food source.

The state transition probability for the *k*-th ant to move from node *i* to the next node *j* can be expressed as

(1) $$\begin{array}{c} P_{ij}^{k}\left(t\right)=\frac{{\left[\tau_{ij}\left(t\right)\right]}^{\alpha }{\left[\eta_{ij}\left(t\right)\right]}^{\beta }}{\sum_{l=\text{allowed}}{\left[\tau_{il}\left(t\right)\right]}^{\alpha }{\left[\eta_{il}\left(t\right)\right]}^{\beta }}, \end{array}$$

where *τ*_*ij*_(*t*) is the pheromone level on a path, *η*_*ij*_(*t*) is the expectation (visibility) for an ant to move from node *i* to the next node *j*, *α*and*β* are the heuristic factors representing pheromone level and visibility, respectively, and *t* is the batch of ant colony, i.e., the number of iterations of the algorithm.

When the colony completes a search task, the pheromone level on each path can be adjusted by the following rules:

(2) $$\begin{array}{c} \tau_{ij}\left(t+1\right)=\left(1-\rho \right)\tau_{ij}\left(t\right)+△\tau_{ij}\left(t\right), \end{array}$$

(3) $$\begin{array}{c} △\tau_{ij}\left(t\right)=\overset{N}{\underset{k=1}{\sum }}△\tau_{ij}^{k}\left(t\right), \end{array}$$

where, if the *k*-th ant passes by a path, then △*τ*_*ij*_^*k*^(*t*)=*Q*/*L*^*k*^(*t*); otherwise, △*τ*_*ij*_^*k*^(*t*)=0, *Q* is the pheromone intensity, *L*^*k*^(*t*) is the total length of the paths traversed by the ants in the current search, *ρ* ∈ [0,1) is the pheromone volatilization coefficient, and *N* is the total number of ants moving out of the nest in the current batch.

The following key problems were solved to design a fault diagnosis plan based on the ant colony classification algorithm.

#### 2.3.1. Generation of Paths and Nodes

**Figure 3:**
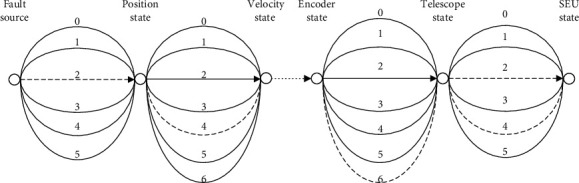
Fault diagnosis as the path search by the ant colony.

Based on the principle of the ACO, the first node was defined as the fault source and the other nodes as attributes. Each path represents the eigenvalue of an attribute. If the eigenvalue is zero, then the attribute is not considered. The other values have the same meaning as the element values of fault eigenvector *E*. When an ant reaches the last node, it means the food source has been discovered. The vector composed of the paths stands for a diagnosis rule. In this way, the fault diagnosis can be described as the path search by the ant colony (Figure 3).

#### 2.3.2. Establishment of Evaluation Function for Classification Performance

The performance of each diagnosis rule generated by the paths traversed by the ants can be quantified by classification precision:

(4) $$\begin{array}{c} f\left(t\right)=\frac{n_{TP}\left(t\right)}{n_{TP}\left(t\right)+n_{FN}\left(t\right)}\cdot \frac{n_{TN}\left(t\right)}{n_{FP}\left(t\right)+n_{TN}\left(t\right)}, \end{array}$$

where *t* is the number of iterations of the algorithm, *n*_*TP*_(*t*) is the number of samples, which should belong to a class, allocated to that class, *n*_*FP*_(*t*) is the number of samples, which should belong to a class, allocated to another class, and *n*_*FN*_(*t*) is the number of samples, which should not belong to a class, allocated to that class. Then, *n*_*TN*_(*t*)/*n*_*FP*_(*t*)+*n*_*TN*_(*t*) was defined as the specificity, i.e., the proportion of the samples, which should not belong to a class, allocated to another class. Obviously, *f* ∈ [0,1], and the closer the *f* is to 1, the more accurate the class judged by the diagnosis rule.

#### 2.3.3. Determination of Path Visibility

The visibility of the path represented by the *j*-th eigenvalue *V*_*ij*_ of the *i*-th attribute *A*_*i*_ can be expressed as the information entropy of the attribute eigenvalue:

(5) $$\begin{array}{c} \eta_{ij}=\frac{\log_{2}\;K-H\left(W|A_{i}=V_{ij}\right)}{\sum_{i=1}^{a}\sum_{j=1}^{b_{i}}\log_{2}\;K-H\left(W|A_{i}=V_{ij}\right)}, \end{array}$$

where *H*(.) is the entropy of the attribute eigenvalue, *K* is the number of classes, *W* is the class attribute, *a* is the number of fault attributes, and *b*_*i*_ is the number of eigenvalues of the *i*-th attribute. Then, the information entropy can be expressed as

(6) $$\begin{array}{c} H\left(W|A_{i}=V_{ij}\right)=-\overset{K}{\underset{\omega =1}{\sum }}\left\{P\left(\omega |A_{i}=V_{ij}\right)\log_{2}\;P\left(\omega |A_{i}=V_{ij}\right)\right\}, \end{array}$$

where *P*(*ω*|*A*_*i*_=*V*_*ij*_) is the empirical probability that *A*_*i*_=*V*_*ij*_ belongs to class *ω*; *i*=1 ~ *a*; and *j*=1 ~ *b*_*i*_.

As shown in formulas (5) and (6), the greater the entropy of the eigenvalue on a path, the more uniform the distribution of the attribute eigenvalue across different classes, the more difficult it is to judge fault class by the attribute eigenvalue, and the lower the visibility of the node. Under the same classification performance, the fewer the attributes or conditions in a diagnosis rule, the stronger the generalization of the rule. The search for the optimal diagnosis rule aims to find the most generalizable rule. Therefore, the path with an eigenvalue of zero is the most visible one.

#### 2.3.4. Initialization and Updation of the Path Pheromone

The pheromone is evenly distributed on each path:

(7) $$\begin{array}{c} \tau_{ij}\left(t=0\right)=\frac{1}{\sum_{i=1}^{a}b_{i}}, \end{array}$$

where *i*=1 ~ *a* and *j*=1 ~ *b*_*i*_.

Once the ants have passed through a path, the pheromone on that path can be updated by

(8) $$\begin{array}{c} \tau_{ij}\left(t+1\right)=\tau_{ij}\left(t\right)+\tau_{ij}\left(t\right)f\left(t\right), \end{array}$$

where *f* is the classification performance score of samples by the diagnosis rule. The higher the *f*, the more the pheromones released by the ants on the path.

#### 2.3.5. Calculation of State Transition Probability

The state transition probability of the ants passing by the path represented by the *j*-th eigenvalue of the *i*-th attribute can be calculated by

(9) $$\begin{array}{c} p_{ij}\left(t\right)=\frac{\eta_{ij}\tau_{ij}\left(t\right)}{\sum_{l=1}^{a}\eta_{il}\tau_{il}\left(t\right)}, \end{array}$$

where *i*=1 ~ *a* and *j*=1 ~ *b*_*i*_. The greater the *τ*_*ij*_, the more the ants have passed through the path and the better the classification performance for the diagnosis rule containing that condition. Since the product between *τ*_*ij*_ and *η*_*ij*_ is the numerator, the greater the *p*_*ij*_ and the more likely it is for the ants to pass by that path.

### 2.4. Expert System for In-Orbit Handling

**Figure 4:**
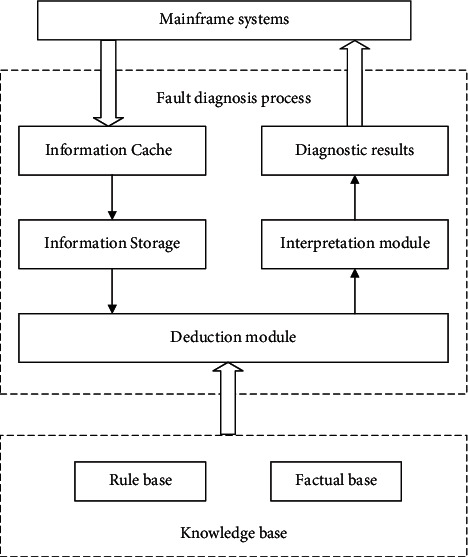
Block diagram of the expert system for in-orbit handling.

When professionals are absent, the fault cannot be diagnosed timely in the field. Then, the known fault phenomenon would be imported to the expert system to diagnose the position of the faulty device. During in-orbit operations, the servo system is, for the most of time, unattended. Thus, the traditional human-computer system was discarded, and the expert system was designed as shown in Figure 4. The proposed expert system mainly encompasses the following: a buffer zone recording the fault phenomenon; a knowledge base containing facts, heuristic rules, and problem-solving rules; an interpretation module for application rules; a reasoning module controlling the sequence of rules.

## 3. Implementation Steps

### 3.1. Fault Diagnosis and Classification Approach

By the ant colony classification algorithm, only one ant is dispatched at a time. Once the ant passes by a path, a condition is added to the corresponding diagnosis rule. If the rule can enhance the classification performance, it will be selected; otherwise, it will be discarded. The latter case is equivalent to the situation that the eigenvalue of the path is set to zero. The main steps of fault diagnosis and classification are as follows:

  Step 1 . Initialize the parameters: solve the visibility of each path by formulas (5) and (6), initialize the pheromone distribution on each path by formula (7), send the first ant from the fault source, and randomly generate an initial path.
  Step 2 . Set the number of iterations as *t* = 1.
  Step 3 . Once the ant passes by the *i*-th path, add a condition to the corresponding diagnosis rule and apply the rule to classify the samples. Then, compute the current classification precision *f*_*i*_(*t*). If *f*_*i*_(*t*) ≤ *f*_*i*−1_(*t*), remove the condition from the rule, i.e., the ant chooses to move along the path with an eigenvalue of zero; otherwise, update the classification precision. After the ant has passed through a path, obtain the final classification precision *f*_*a*_(*t*), which is the given initial evaluation score.
  Step 4 . According to *f*_*a*_(*t*), modify the pheromone of the passed path by formula (8). Combined with visibility, compute the state transition probability *p*_*ij*_(*t*) of all paths and generate the path for the next ant in the light of *p*_*ij*_(*t*).
  Step 5 . If *f*_*a*_(*t*) is equal to or greater than the given classification precision *ε*_0_, go to Step 6; otherwise, jump to Step 7.
  Step 6 . Record the diagnosis rule found by the ant, classify the remaining samples in the fault library, and remove the samples, which should belong to a class, allocated to that class by that rule. Besides, reinitialize the pheromone and visibility of each path, and then generate the path for the next ant.
  Step 7 . If the number of samples, which should belong to a class, allocated to that class reaches the preset requirement, or the algorithm reaches the maximum number of iterations *t*_max_, then terminate the algorithm and record the final data; otherwise, set *t*=*t*+1, return to Step 3, and send a new ant to search for the next diagnosis rule.

### 3.2. In-Orbit Fault Handling by the Expert System

Since the in-orbit operations of the servo system is remotely controlled, it is impossible for the ground to monitor and control the working condition and mode of the system in real time. To ensure that ground control overrides the autonomous software handling, the in-orbit fault handling of the expert system was controlled by two working modes (entering fault handling mode and exiting fault handling mode) and one working sign (in-orbit autonomous handling sign). In addition, three working states were employed to indicate the current fault handling by the servo system, including fault handling underway, fault handling completed, and fault handling failed.

**Figure 5:**
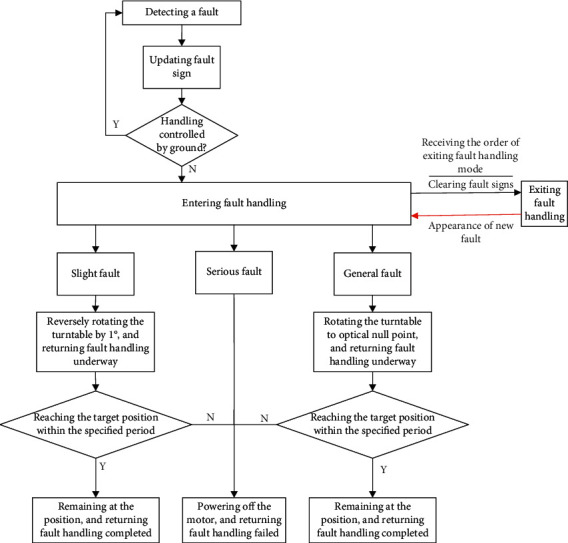
In-orbit fault handling flow of the expert system.

As shown in Figure 5, the expert system is divided into three parts: fault identification and classification, fault handling, and fault exiting.

#### 3.2.1. Fault Identification and Classification

Whether the servo system is faulty and, if so, what is the type of system fault were recognized by the fault diagnosis rules generated by ant colony fault diagnosis and classification. After recognizing a fault, the servo system would firstly update the fault sign and check if the working sign is in-orbit autonomous handling. If yes, the working mode of the servo system would be switched to entering fault handling mode.

#### 3.2.2. Fault Handling

The servo system could handle the recognized fault in four different ways:

1. *Slight Fault*. The servo system switches the working mode into entering fault handling mode and updates the working state to fault handling underway. The faulty position would be rotated by 1° opposite to the moving direction of the system. After the rotation is complete, the system would return faulty handling completed and continue to execute its task after two more minutes.
2. *General Fault*. The servo system switches the working mode into entering fault handling mode and updates the working state to fault handling underway. Then, the system would rotate to the optical null point. After reaching that position, the system would remain there, return fault handling completed, and wait for orders from the ground.
3. *Serious Fault*. The servo system switches the working mode into entering fault handling mode and updates the working state to fault handling failed. Then, the system would power off the motor and wait for orders from the ground.
4. *Fault Handling Failed*. If the servo system fails to rotate to the specific position within a specified period after encountering a slight or general fault, it would update the working state to fault handling failed. Then, the system would power off the motor and wait for orders from the ground.

#### 3.2.3. Fault Exiting

Upon receiving the order of exiting fault handling mode, the servo system would enter that mode and clear all fault signs, regardless of the current stage of fault handling.

## 4. Simulation

Taking the fault diagnosis of velocity state, torque state, and SEU state of the servo system for example, this paper calls the ACO to search for the optimization rules capable of accurately predicting these faults and simulates the in-orbit fault handling by the expert system.

The simulation parameters of the ACO were set as follows: the initial evaluation value *f*_0_(*t*)=0, preset classification precision *ε*_0_=0, and preset maximum number of iterations *t*_max_=200, which represents the maximum batch of ants being dispatched.  Table 2 reports the simulation results.

The simulation results show that the ACO can find diagnosis rules with a few conditions and a high classification precision within a limited number of iterations and predict the fault samples belonging to these classes correctly. Meanwhile, the expert system can correctly handle the predicted faults.

## 5. Conclusions

After building a fault library of the 2D tracking servo system for space, this paper calls the ACO to optimize the fault diagnosis rules of the servo system and it eliminates the inherent redundant attributes of fault attribute representation, so as to achieve the purpose of using fewer condition items to more accurately determine the fault category. During the in-orbit operations of the servo system, the in-orbit expert system can precisely judge the fault type and handle the detected faults, using a limited number of software and hardware resources. Experiments show that the ACO algorithm combined with the in-orbit processing expert system has the ability to quickly search and the fault diagnosis rules after ant colony optimization have the advantages of fewer rules and condition items, high diagnostic accuracy, high prediction success rate, less resource occupation, and fast processing. Therefore, the fault diagnosis and processing method based on the combination of ACO algorithm and in-orbit processing expert system has broad application prospects in the field of aerospace fault diagnosis.

## Figures and Tables

**Table 1:** Relationship between telemetered values and fault phenomena.

Fault phenomena	Telemetered value 0 × 00	Telemetered value 0 × 03	Telemetered value 0 × 0C	Telemetered value 0 × 30	Telemetered value 0 × C0
*e*_1_ position state	Normal	Reverse limit of azimuth axis	Forward limit of azimuth axis	Reverse limit of pitch axis	Forward limit of pitch axis
*e*_2_ velocity state	Normal	Reverse limit of azimuth axis	Forward limit of azimuth axis	Reverse limit of pitch axis	Forward limit of pitch axis
*e*_3_ current state	Normal	Reverse limit of azimuth axis	Forward limit of azimuth axis	Reverse limit of pitch axis	Forward limit of pitch axis
*e*_4_ torque state	Normal	Reverse limit of azimuth axis	Forward limit of azimuth axis	Reverse limit of pitch axis	Forward limit of pitch axis
*e*_5_ motor temperature state	Normal	Abnormal temperature of pitch axis	Abnormal temperature of pitch axis	Abnormal temperature of pitch axis	Abnormal temperature of pitch axis
*e*_6_ tracking precision state	Normal	Reverse limit of azimuth axis	Forward limit of azimuth axis	Reverse limit of pitch axis	Forward limit of pitch axis
*e*_7_ communication state	Normal	Abnormal communication	Abnormal communication	Abnormal communication	Abnormal communication
*e*_8_ AD signal collection state	Normal	Abnormal phase A of azimuth axis	Abnormal phase B of azimuth axis	Abnormal phase A of pitch axis	Abnormal phase B of pitch axis
*e*_9_ encoder state	Normal	Abnormal encoder precision of azimuth axis	Encoder calculation fault of azimuth axis	Abnormal encoder precision of pitch axis	Encoder calculation fault of pitch axis
*e*_10_ guide telescope state	Normal	Abnormal data	Abnormal gray value	Abnormal AD signal collection	Abnormal communication
*e*_11_ SEU state	Normal	Abnormal calculation data of azimuth axis	Abnormal mode sign of azimuth axis	Abnormal calculation data of pitch axis	Abnormal mode sign of pitch axis

**Table 2:** Fault diagnosis and handling results.

Fault type	Number of rules	Number of iterations under the rule	Classification precision under the rule *f*_*a*_(*t*)	Handling result
Velocity state	5	23	0.9341	Handled as a serious fault
			0.8213	Handled as a serious fault
			0.8559	Handled as a serious fault
			0.9411	Handled as a serious fault
			0.8729	Handled as a serious fault

Torque state	5	45	0.8579	Handled as a general fault
			0.9590	Handled as a general fault
			0.8761	Handled as a general fault
			0.9322	Handled as a general fault
			0.9121	Handled as a serious fault

SEU state	4	16	0.8242	Handled as a slight fault
			0.8521	Handled as a slight fault
			0.8821	Handled as a serious fault
			0.9415	Handled as a serious fault

## Data Availability

The data used to support the findings of this study are available from the corresponding author upon request.
